# 834 Effectiveness of Hemostatic Net in Non-meshed Partial-thickness Skin Grafts

**DOI:** 10.1093/jbcr/iraf019.365

**Published:** 2025-04-01

**Authors:** Alberto De Anda Coronado, José ignacio Fonseca-Sada, Cynthia Minerva González Cantú, Yanko Castro Govea, Everardo Valdés Flores

**Affiliations:** Hospital Universitario Dr. José Eleuterio González Universidad Autónoma de Nuevo León; Hospital Universitario Dr. José Eleuterio González; Hospital Universitario Dr. José Eleuterio González Universidad Autónoma de Nuevo León; Hospital Universitario Dr. José Eleuterio González Universidad Autónoma de Nuevo León; Hospital Universitario Dr. José Eleuterio González Universidad Autónoma de Nuevo León

## Abstract

**Introduction:**

Skin grafting is an indispensable technique for covering skin defects and wounds, and it is part of the multiple reconstructive options of every plastic surgeon. Skin grafts are used as a reconstructive option in a wide variety of clinical scenarios such as burns, reconstruction of burn sequelae, reconstruction of hypertrophic scars or release of contractile scars, coverage of skin defects after oncologic resections, congenital skin deficiencies, hair restoration, among others. Skin grafts are generally classified into partial-thickness and full-thickness. When the graft includes only a portion of the dermis it is called a partial-thickness, and when it consists of the entire dermis it is known as a full-thickness graft. The most common causes of skin graft failure are hematoma, seroma, infection, and shear forces. The hemostatic net has been used in cosmetic procedures to reduce the risk of postoperative hematoma. This is achieved by causing total occlusion of the surgical area between the dissection planes preventing fluid accumulation.

**Methods:**

For each patient enrolled, we performed a non meshed partial-thickness skin graft area, using hemostatic net with absorbable suture, and an area of partial-thickness skin graft meshed. The percentage of graft integration was determined by evaluating the graft 14 and 21 days after surgery, by visual evaluation of the graft.

**Results:**

A total of 10 adult patients were enrolled. Twelve burn areas were treated, all of them managed with non-meshed partial thickness skin graft, using hemostatic net, and an area of meshed partial thickness skin graft. A group of 15 plastic surgery residents visually evaluated the grafts 14 and 21 days after surgery. In all 12 areas, the grafts were 100% integrated, with no seroma or hematoma.

**Conclusions:**

We showed promising results in our study, by applying the technique initially described for use in cosmetic procedures comparing against the classic method of meshed skin graft. However, comparative studies with a larger population are necessary to increase the reliability of this procedure.

**Applicability of Research to Practice:**

Based on our results, we can affirm that the use of the hemostatic net reduces complications such as seromas and hematomas in patients who underwent skin grafting. Its applicability reduces costs, reduces hospitalization times, and improves patient prognosis.

**Funding for the Study:**

N/A

